# The prognostic and predictive value of tumor-infiltrating lymphocytes and hematologic parameters in patients with breast cancer

**DOI:** 10.1186/s12885-018-4832-5

**Published:** 2018-10-01

**Authors:** Kwan Ho Lee, Eun Young Kim, Ji Sup Yun, Yong Lai Park, Sung-Im Do, Seoung Wan Chae, Chan Heun Park

**Affiliations:** 1 Department of Surgery, Kangbuk Samsung Hospital, Sungkyunkwan University School of Medicine, 29 Saemunan-ro, Jongno-gu, Seoul, 03181 South Korea; 2 Department of Pathology, Kangbuk Samsung Hospital, Sungkyunkwan University School of Medicine, 29 Saemunan-ro, Jongno-gu, Seoul, 03181 South Korea

**Keywords:** CD8, FOXP3, Tumor-infiltrating lymphocyte, Breast cancer, Lymphocyte-monocyte ratio

## Abstract

**Background:**

Carcinogenesis and tumor growth are associated with chronic inflammation and the host immune system. Here, we investigated the clinical significance and relationship between tumor-infiltrating lymphocytes (TILs) and hematologic parameters in patients with breast cancer.

**Methods:**

Invasive ductal breast cancer patients (*N* = 145) who underwent surgery were retrospectively evaluated. Samples were obtained using a core needle biopsy for CD8+, FOXP3+ TIL assessment. Blood lymphocytes, neutrophils, monocytes, and platelets were obtained by peripheral venous punctures.

**Results:**

CD8 + TILs were significantly associated with absolute lymphocyte count (ALC) and the absolute monocyte count (AMC). Low LMR (ALC/AMC) (cut-off - 5.3, range = 0.73–12.31) was associated with poor overall survival (OS) (*p* = 0.010), disease-free survival (DFS) (*p* = 0.005). However, in subgroup analysis, LMR did not have any value as a prognostic factor in HER2-positive breast cancers. TILs had different prognostic impacts across breast cancer subtypes, although they were not statistically significant. The treatment response after NAC tended to improve in breast cancer patients with high FOXP3+ TILs, low NLR (neutrophil count/ALC) (FOXP3 *p* for trend = 0.006, NLR *p* for trend = 0.063).

**Conclusions:**

A relevance between TILs and hematologic parameters in breast cancer was demonstrated. The influence of the immune system on breast cancer progression may differ by subtype.

**Electronic supplementary material:**

The online version of this article (10.1186/s12885-018-4832-5) contains supplementary material, which is available to authorized users.

## Background

Over the past several decades, it has been demonstrated that carcinogenesis and tumor growth are associated with chronic inflammation and the host immune system [1]. Thus, immunomodulating therapies have emerged as effective and novel therapeutic strategies [2–4]. Peripheral blood parameters have been studied as a prognostic factor whose extraction is user friendly and less expensive in the measurement of systemic immunity [5–7]. In addition, there is a method for examining lymphocyte infiltration into cancer tissue in relation to local immunity, which is reported to be related to the prognosis of various malignancies [8–10]. However, little is known about the relationship between local and systemic immune responses [7, 11].

Tumor-infiltrating lymphocytes (TILs) have been studied as an indicator of tumor inflammation, and it has been reported that TIL subsets have their own roles in breast cancer progression. Several studies have reported that CD8+ TILs are associated with good clinical prognoses in breast cancer patients [12, 13]. Moreover, the forkhead box protein 3 (FOXP3) plays an important role in the generation of regulatory T cells (Treg), but the precise role, function, and prognostic abilities of FOXP3 in breast cancer have yet to be established [14–16].

The chronic systemic inflammatory response has been continuously studied in relation to the progression and prognosis of breast cancer [17]. Peripheral blood-based parameters have been studied as factors that reflect a host’s immune response, and it has been suggested that circulating white blood cells, resulting in a change in the proportions of neutrophils, lymphocytes, and monocytes, may be associated with systemic inflammatory responses [18]. The relationships between cancer prognosis and absolute monocyte count (AMC), absolute lymphocyte count (ALC), lymphocyte-to-monocyte ratio (LMR), neutrophil-to-lymphocyte ratio (NLR), and platelet-to-lymphocyte ratio (PLR) have been studied in various cancers [19–22]. In patients with breast cancer, Ni et al. have reported that an elevated LMR is a favorable prognostic factor following neoadjuvant chemotherapy (NAC) [23]. Moreover, Azab et al. demonstrated that NLR is superior in predicting long-term outcomes over PLR [24].

In this study, we attempted to evaluate the association between local and systemic immune responses by comparing the correlation between TILs and peripheral blood hematologic parameters, which have not yet been studied in breast cancer. The prognostic effect of immune-related markers was analyzed by breast cancer subtypes. Moreover, we estimated a predictive value of immune-related markers for response to NAC.

## Methods

### Patients

Data on 232 invasive ductal breast cancer patients who underwent surgery at Sungkyunkwan University, Kangbuk Samsung Hospital between December 2005 and September 2015 were retrospectively evaluated. The exclusion criteria were as follows: (i) patients with distant metastases at initial presentation, bilateral breast carcinoma, and male breast carcinoma; (ii) patients with comorbidities that affected levels of inflammatory parameters, including infection, hematological disorders, collagen disease; and (iii) patients without sufficient formalin-fixed and paraffin-embedded resection tissue for immunostaining. Ultimately, 145 patients were eligible for analysis and were reviewed retrospectively. Clinical information (such as age, menopausal status, tumor size, lymph node status, histologic grade, lymphovascular invasion (L/V invasion), estrogen receptor (ER) status, progesterone receptor status, human epidermal growth factor receptor 2 (HER2) status, and primary treatment information (including surgery, radiotherapy, and chemotherapy) were extracted from the medical records. Histological grade was defined according to the Elston and Ellis classification [25].

For patients who received NAC, Response Evaluation Criteria in Solid Tumors (RECIST ver 1.1) was used to assess the response to treatment [26]. In this study, progressive disease (PD) and stable disease (SD) were defined as no response (NR) to NAC treatment. The tumor responses after NAC were classified into three groups: pathologic complete response (pCR), partial response (PR), and NR. pCR was defined as the absence of tumor cells or absence of persistent in situ disease and negative axillary lymph nodes. All patients with NAC were treated by anthracycline-based regimen. Of the 44 patients, the AD regimen, consisting of doxorubicin (50 mg/m^2^) and docetaxel (75 mg/m^2^) on day 1 every 3 weeks for 4 cycles, was used in 27 patients (61%); the AC regimen, consisting of doxorubicin (60 mg/m^2^) and cyclophosphamide (600 mg/m^2^) on day 1 every 3 weeks for 4 cycles, was used in 2 patients(5%); and sequential ACT, comprising 4 cycles of AC followed by 4 cycles of docetaxel (100 mg/m^2^), was used in 15 patients (34%). The patients with HER2+ breast cancer received trastuzumab triweekly (6 mg/kg) for 12 months. All patients with NAC underwent breast surgery about 3–4 weeks after the last chemotherapy cycle. This study was approved by the Institutional Review Board of Kangbuk Samsung Hospital, the Sungkyunkwan University of Korea, on 8 August 2017 (KBSMC 2017–07-047).

### Follow-up

All patients underwent a physical examination at 3-month intervals after surgery, breast ultrasonography, mammography and chest CT at 6-month intervals, and bone scan, breast MRI at 1-year intervals. The last follow-up date was April 30, 2017, for all of the available patients. Disease-free survival (DFS) was defined as the interval between the date of diagnosis for breast cancer and the date of having evidence of recurrent events (i.e., invasive recurrence at any sites, or a new invasive breast cancer in the contralateral breast) or death of any cause. Overall survival (OS) was calculated from the date of diagnosis to death (of any causes) or the date of the last follow-up.

### Blood sample analysis

All blood samples were obtained by peripheral venous puncture 7 days prior to any treatment for breast cancer. The blood samples were placed in tubes containing ethylenediaminetetraacetic acid (EDTA) and immediately sent for analysis. In our institution, all blood sampling before surgery or chemotherapy was performed after 8 h of fasting. Hematologic parameters (i.e., the number of lymphocytes, neutrophils, monocytes, and platelets) were counted by an automated hematology analyzer (XN-5000, Sysmex, Kobe, Japan). LMR was calculated by dividing the ALC by the AMC. NLR was defined as the absolute neutrophil count (ANC) divided by the ALC, and PLR as the absolute platelet count divided by the ALC.

### Immunohistochemical (IHC) staining

**Fig. 1:**
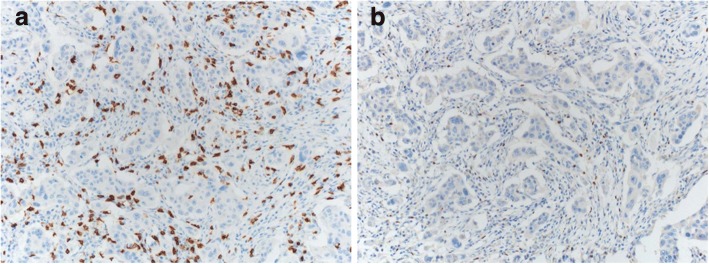
Tumor CD8+ and FOXP3+ expression as assessed with IHC in breast cancer. **a** CD8 IHC stain in breast carcinoma highlights abundant CD8+ T lymphocytes (200X magnification); (**b**) FOXP3 IHC stain in breast carcinoma highlights FOXP3+ lymphocytes (200X magnification). *FOXP3+* forkhead box protein 3, *IHC* immunohistochemistry

All patient samples were obtained before any treatment modality using core needle biopsies, and samples were fixed in 10% formaldehyde solution and then embedded in paraffin. The formalin-fixed paraffin-embedded specimens were cut into 3 μm thick sections for IHC staining. The sections were dehydrated and deparaffinized in xylene and then rehydrated in a graded series of alcohol solutions. Primary antibodies used are as follows: ER (1:200; SP1; Lab Vision Corporation, Fremont, CA, USA), progesterone receptor (1:200; PgR636; Dako, Glostrup, Denmark), HER2 (1:1; clone 4B5; Ventana Medical Systems, Tucson, AZ, USA), Ki-67 (1:200; MIB-1; Dako), CD8 (1:100; clone C8/144B; Dako), and FOXP3 (1:100; clone 236A/E7; Abcam, Cambridge, UK). Immunostaining was performed using a compact polymer method (Bond Intense Detection Kit; Leica Biosystems, Newcastle upon Tyne, UK). The primary antibodies were detected with Dako EnVision+ Systems (HRP; DakoCytomation, Glostrup, Denmark), according to the manufacturer’s instructions. The Dako EnVision+ Detection Systems, Peroxidase/DAB (DakoCytomation, Glostrup, Denmark), was used to perform chromogenic visualization. The slides were then counterstained with hematoxylin, and coverslips were applied. To evaluate CD8 and FOXP3 TILs, five stained areas were selected, and the number of TILs in the stroma surrounding the stained cancer cells was measured quantitatively in each field under 200× magnification (Fig. 1). All slides were examined and scored by a board-certified pathologist (D.S-I.) who was blinded to the patient’s clinicopathological data.

### Statistical analyses

All statistical analyses were performed using IBM SPSS version 24.0 software (IBM Corp., Armonk, NY, USA). Associations among variables were evaluated using Fisher’s exact test or the Chi-square test for category variables and one-way analysis of variance (ANOVA) or Student’s *t*-test or Spearman rank correlation for continuous variables. The Kaplan-Meier method was used to compare DFS and OS. To evaluate the effect of the prognostic variables, univariate and multivariate analyses were performed using Cox’s proportional hazards model.

## Results

The baseline characteristics of the patients are shown in Table 1. All patients were female with a median age of 49 years at the time of diagnosis. Forty-four of 145 patients (30%) received NAC, 95 (66%) received adjuvant chemotherapy, and 6 (4%) did not receive chemotherapy. A total of 35 of 145 (24%) patients underwent breast conserving surgery and 110 (76%) underwent mastectomy, with sentinel lymph node biopsy or axillary lymph node dissection. The cut-off points for the TILs and hematologic parameters were based on the median value of each factor, which were ALC (1.94 × 10^9^/L (range, 0.62–3.52 × 10^9^/L)), AMC (0.36 × 10^9^/L (range, 0.18–0.85 × 10^9^/L)), ANC (3.30 × 10^9^/L (range, 1.48–10.1 × 10^9^/L)), NLR (1.72 (range 0.76–25.36)), LMR (5.3 (range 0.73–12.31)), PLR (8.24 (range 0.13–2.5)), CD8 (30% (range 0–82%)), and FOXP3 (5.2% (range 0–41%)).

**Table 1:** Basic characteristics of the enrolled patients

Variables	No. (%)
Age (years)	
≤ 50	77 (53)
> 50	68 (47)
Pathologic T stage	
1	50 (35)
2–4	95 (65)
Pathologic N stage	
0	54 (37)
1–3	91 (63)
Histologic grade	
1–2	92 (64)
3	53 (36)
Lymphovascular invasion	
Absent	86 (59)
Present	59 (41)
Type of surgery	
Breast conserving surgery	35 (24)
Mastectomy	110 (76)
Estrogen receptor	
Negative	64 (44)
Positive	81 (56)
Progesterone receptor	
Negative	87 (60)
Positive	58 (40)
HER2	
Negative	108 (75)
Positive	37 (25)
Chemotherapy	
Neoadjuvant	44 (30)
Adjuvant	95 (66)
No	6 (4)
Adjuvant radiotherapy	
No	81 (56)
Yes	64 (44)

HER2, human epidermal growth factor receptor 2

### Correlation between TILs and hematologic parameters

CD8+ TILs were significantly correlated with the FOXP3+ TILs (*r* = 0.41, *p* <  0.001). The relationship between TILs and hematologic markers, which we considered to be of interest in this study, also showed some statistical significance. CD8+ and AMC were positively correlated (*r* = 0.22, *p* = 0.010), while CD8+ and ALC showed a negative relationship (*r* = − 0.24, *p* = 0.004). Therefore, LMR, which was calculated by these two factors, also showed a significant correlation with CD8+ (*r* = 0.20, *p* = 0.019). In contrast, FOXP3+ showed no statistically significant association with any hematologic marker (Table 2).

**Table 2:** Correlation between hematologic and TILs

	FOXP3	AMC	ALC	ANC	LMR	NLR	PLR
CD8							
r	0.41	0.22	−0.24	0.04	0.20	−0.06	− 0.12
*p*	< 0.001*	0.010*	0.004*	0.614	0.019*	0.518	0.176
FOXP3							
r		−0.11	0.01	0.08	−0.11	− 0.09	−0.01
*p*		0.204	0.922	0.362	0.200	0.307	0.891

Partial Correlation Coefficients by Spearman

*AMC* absolute monocyte count, *ALC* absolute lymphocyte count, *ANC* absolute neutrophil count, *LMR* lymphocyte-to-monocyte ratio, *NLR* neutrophil-to-lymphocyte ratio, *PLR* platelet-to-lymphocyte ratio, *FOXP3* forkhead box protein 3

*p < 0.05

### Survival analyses based on CD8+, FOXP3+ TILs, LMR, and clinicopathological characteristics

**Fig. 2:**
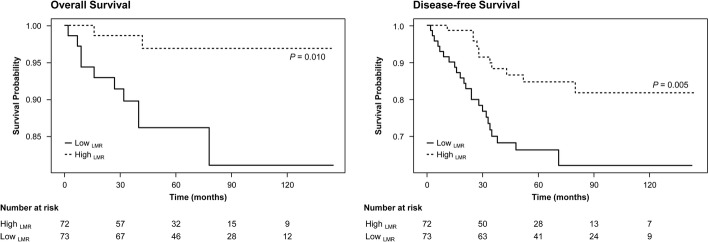
Kaplan–Meier survival analysis of baseline hematologic parameter (LMR) in 145 breast cancer patients. *LMR* lymphocyte to monocyte ratio

After a mean follow-up of 71 months (range, 2–145 months), 34 (23%) had cancer recurrence and 12 (8%) died among the 145 patients. Kaplan–Meier survival curve analysis showed that the DFS and OS of breast cancer patients with an high LMR (≥5.3) was significantly longer than those with an low LMR (< 5.3) (DFS, *p* = 0.005; OS, *p* = 0.010) (Fig. 2). On the other hand, PLR, NLR and TILs did not show any association with prognosis (Additional file 1: Figure S1).

When the relationship between DFS and clinicopathological variables was examined using univariate analysis, large tumor size (*p* = 0.029), lymph node metastasis (*p* = 0.026), L/V invasion (*p* <  0.001) and low LMR (*p* = 0.008) were associated with a lower DFS. The multivariate analysis that controlled for all factors with associations emerging from the univariate analysis (*p* <  0.2) revealed that L/V invasion, HER2 status, and LMR status were independent predictors of DFS (L/V invasion - hazard ratio [HR] 3.29 (1.58–6.84), *p* = 0.014; HER2 positivity-HR 2.38 (1.11–5.10), *p* = 0.025; high LMR - HR 0.43 (0.20–0.90), *p* = 0.024). TILs were not prognostically significant with the survival analysis (Table 3). The LMR status was the only independent predictor of OS (HR 0.17 (0.04–0.80), *p* = 0.025) (Additional file 2: Table S1).

**Table 3:** Univariate and multivariate Cox regression analyses of TILs, LMR, and clinicopathological characteristics for survival in patients with breast cancer

Variable	Disease-free survival			
	Univariate analysis		Multivariate analysis	
	HR (95% CI)	*p*	HR (95% CI)	*p*
Age (> 50 vs. ≤50)	0.86 (0.44–1.70)	0.673		
T stage (>T1 vs. T1)	2.53 (1.10–5.83)	0.029	2.16 (0.92–5.09)	0.079
N stage (>N0 vs. N0)	2.46 (1.11–5.45)	0.026	1.70 (0.75–3.86)	0.204
Histologic grade (G3 vs. <G3)	1.25 (0.63–2.48)	0.518		
Lymphovascular invasion (yes vs. no)	3.62 (1.76–7.43)	< 0.001	3.29 (1.58–6.84)	0.001
Estrogen receptor (positive vs. negative)	0.80 (0.41–1.58)	0.526		
Progesterone receptor (positive vs. negative)	0.62 (0.30–1.28)	0.196	0.89 (0.42–1.87)	0.752
HER2 (positive vs. negative)	1.95 (0.96–3.96)	0.063	2.38 (1.11–5.10)	0.025
Type of operation (Mastectomy vs. BCS)	3.14 (0.96–10.27)	0.059	2.38 (0.70–8.12)	0.164
CD8 (high vs. low)	1.04 (0.53–2.05)	0.899		
FOXP3 (high vs. low)	0.97(0.50–1.91)	0.937		
LMR (high vs. low)	0.37(0.18–0.77)	0.008	0.43(0.20–0.90)	0.024

*CI* confidence interval, *HER2* human epidermal growth factor receptor 2, *FOXP3* forkhead box protein 3, *BCS* breast conserving surgery, *LMR*, lymphocyte/monocyte ratio

We also investigated the prognostic impact of the immune-related markers across the 3 breast cancer subtypes: hormone receptor negative/HER2− (TN), hormone receptor positive/HER2− (HR^+^), and hormone receptor positive or hormone receptor negative/ HER2+ (HER2^+^). No statistical significance was found for any parameters of HER2^+^ tumor. On the other hand, low LMR and high ALC were significant predictors of favorable DFS for HR^+^ breast cancer (high ALC HR 0.26, CI 0.03–0.59, *p* = 0.024; high LMR HR 0.13 (0.03–0.59) *p* = 0.008). In TN breast cancer, low AMC was statistically related to a favorable DFS and high LMR was marginally related (high AMC HR 5.18, CI 1.08–24.94, *p* = 0.040; high LMR HR 0.23 (0.05–1.11), *p* = 0.067). Considering that LMR is the value of ALC divided by AMC, ALC in HR^+^ and AMC in TN tumors have prognostic relevance. There was no statistical difference in the prognostic abilities of the TILs in any breast cancer subtype (Table 4).

**Table 4:** Univariate analysis of TILs and the hematologic parameters associated with DFS in each of the breast cancer subtypes

Variable	Disease-free survival					
	HR^+^		HER2^+^		Triple-negative	
	HR (95% CI)	*p*	HR (95% CI)	*p*	HR (95% CI)	*p*
AMC (high vs. low)	1.68 (0.52–5.45)	0.390	0.87 (0.27–2.74)	0.809	5.18 (1.08–24.94)	0.040*
ANC (high vs. low)	0.47 (0.15–1.43)	0.181	0.47 (0.15–1.47)	0.194	1.03 (0.28–3.85)	0.961
ALC (high vs. low)	0.26 (0.08–0.83)	0.024*	0.56 (0.18–1.77)	0.323	0.34 (0.07–1.63)	0.177
NLR (high vs. low)	1.06 (0.36–3.17)	0.911	0.82 (0.26–2.54)	0.729	2.54 (0.63–10.15)	0.188
LMR (high vs. low)	0.13 (0.03–0.59)	0.008*	1.14 (0.36–3.58)	0.828	0.23 (0.05–1.11)	0.067
PLR (high vs. low)	0.87 (0.29–2.59)	0.801	1.08 (0.34–3.42)	0.892	1.49 (0.40–5.56)	0.551
CD8 (high vs. low)	0.91 (0.30–2.71)	0.860	0.65 (0.19–2.19)	0.483	2.75 (0.69–10.99)	0.153
FOXP3 (high vs. low)	0.78 (0.26–2.34)	0.664	0.78 (0.25–2.43)	0.664	1.41 (0.38–5.27)	0.608

*AMC* absolute monocyte count, *ALC* absolute lymphocyte count, *ANC* absolute neutrophil count, *LMR* lymphocyte-to-monocyte ratio, *NLR* neutrophil-to-lymphocyte ratio, *PLR* platelet-to-lymphocyte ratio, *FOXP3* forkhead box protein 3, *HER2*^*+*^ hormone receptor positive or negative/ HER2-positive, *HR*^*+*^ hormone receptor positive/HER2-negative

*p < 0.05

### Correlations between CD8+, FOXP3+ TILs, hematologic parameters, and the response to NAC

**Fig. 3:**
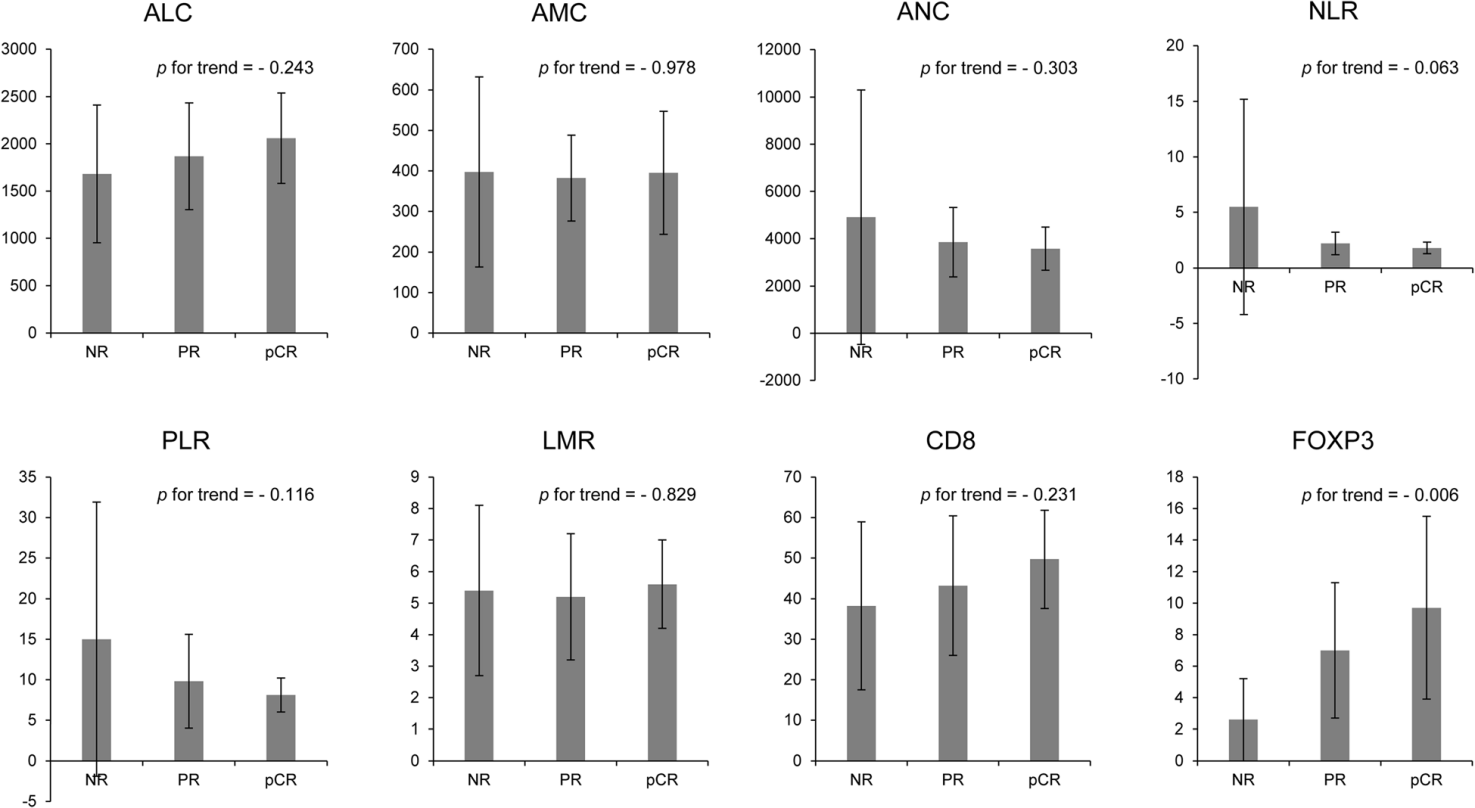
Relationships between TILs, hematologic parameters, and tumor response in patients who underwent neoadjuvant chemotherapy. *ALC* absolute lymphocyte count, *AMC*, absolute monocyte count, *ANC* absolute neutrophil count, *FOXP3+* forkhead box protein 3, *LMR* lymphocyte-to-monocyte ratio, *NLR* neutrophil-to-lymphocyte ratio, *NR* no response, *pCR* pathologic complete response, *PLR* platelet-to-lymphocyte ratio, *PR* partial response

Of the 145 patients, 44 (30%) received NAC. High TILs were associated with the tendency for a better response to chemotherapy, but only FOXP3+ was statistically significant (FOXP3+, *p* for trend = 0.006; CD8+, *p* for trend = 0.231). Among the hematologic parameters, although not significant, there was a tendency for an increased ALC and a decreased ANC to be associated with a better response to NAC. NLR, which was calculated using these two factors, had marginally significant relevance to predict a response to NAC (*p* for trend = 0.063; Fig. 3).

### Association of TILs and hematologic parameters with pathological factors

We compared the levels of hematologic parameters with various pathological factors commonly associated with breast cancer prognosis. In TN breast cancer, all parameters were determined to be lower than in other subtypes, especially the hematologic markers, AMC, ALC, and ANC, that were statistically significant (AMC (*p* = 0.034), ALC (*p* = 0.022), ANC (*p* = 0.041)). Low ANC values were significantly associated with L/V invasion (*p* = 0.006) and tumor histologic grade 3 (*p* = 0.033), and ALC was higher in patients with PR positivity (*p* = 0.015).

## Discussion

The adaptive immune response is related to cancer progression, and it has been thought that TILs could represent tumor immune microenvironments. Developing drugs that could modulate the existing immune response, such as immune checkpoint inhibitor-directed monoclonal antibodies, could be an effective way to limit cancer progression. Similarly, TILs could be used as an immune-related cancer treatment, and several studies are currently being conducted [27, 28]. CD8+ TILs, a subset of TILs, play a role in inducing the death of tumor cells, and Treg cells induce the inactivation of CD8+ TILs. FOXP3 is currently the most commonly used marker of Treg cells. Consistent with this rationale, it has been shown that breast cancer with high CD8+ TILs is associated with a good prognosis and is also associated with achieving high pCR rate in patients receiving NAC [12, 13, 29, 30]. However, the relationship between FOXP3+ TILs and prognosis is unclear as the existing studies present contradictory results [16, 31, 32]. Although this is not yet known, the role of FOXP3 in breast cancer prognosis may be different for each molecular subtype of breast cancer. West et al. reported that high FOXP3+ Treg cells were associated with a favorable outcome only in ER-negative tumors [16]. In another study, FOXP3+ Treg cells expressed in a heterogeneous population of cells that have both regulatory and non-regulatory T-cell functions induce the secretion of heterogeneous cytokines, which play a variety of roles in cancer progression [33]. In this study, neither CD8+ nor FOXP3+ TILs showed a significant relationship with prognosis in breast cancer. However, when analyzed by subtype, high TILs showed a correlation with poor prognosis in TN tumors (CD8-HR 2.75, CI 0.69–10.99; FOXP3-HR 1.41, CI 0.38–5.27) and the opposite tendency in the other breast cancer subtypes, although these results were not statistically significant. In patients who underwent NAC, high TILs were associated with a better response to chemotherapy. These results are consistent with previous studies [29, 30].

It has been recognized that breast cancer is not a local disease but a systemic disease. Therefore, it is thought that the human immune system plays an important role in cancer progression or suppression. Therefore, studies have shown that many inflammatory molecule-based scoring systems are valuable as predictors of cancer prognosis and therapeutic effect [19–24]. For instance, lymphocytes are key immune cells in both humoral and cellular antitumor immune responses, and restrict proliferation and metastasis of tumor cells [34]. A low lymphocyte count has been associated with generalized suppression of the immune system in patients with cancer, and tended to be associated with a worse prognosis [35, 36]. Moreover, neutrophilia is caused by the paraneoplastic activity of a primary tumor and sometimes by the production of granulocyte colony–stimulating factor marrow granulocytic cells due to an interaction between malignant cells and bone [37, 38]. Additionally, monocytes differentiate into tumor-associated macrophages in the tumor and induce the progression of the tumor by producing various cytokines and growth factors that cause angiogenesis and anti-immune responses [39]. Platelets are associated with poor cancer prognosis because they induce the platelet-derived growth factor, vascular endothelial growth factor, and platelet factor 4, which can lead to tumor cell proliferation or invasion into other cells [40, 41]. In this study, among the various hematologic parameters, only LMR was proven to be an independent predictor of OS and DFS in breast cancer patients (OS, HR = 0.17 (0.04–0.80), *p* = 0.025; DFS, HR = 0.43 (0.20–0.90, *p* = 0.024.). However, in the subgroup analysis, LMR had no value in predicting DFS in HER^+^ breast cancers, which was consistent with previous studies [42]. Interestingly, the lymphocyte count had a significant effect on DFS in HR^+^ subtype (HR 0.26, CI 0.03–0.59, *p* = 0.024), whereas the monocyte count had a significant effect on DFS in the TN subtype (HR 5.18, CI 1.08–24.94, *p* = 0.040), resulting in a significant prognostic value for LMR. These results suggest that the effect of the human immune response on cancer progression may be different according to each molecular subtype of breast cancer.

The mechanisms by which LMR has some prognostic relevance in breast cancer patients can be assumed to be related to tumor infiltrating immune cells, such as TILs, or tumor-associated macrophages. Circulating lymphocytes affect TIL formation and may be associated with an immune response in the tumor. Tumor-associated macrophages also cause monocytes to enter the tumor, secrete multiple cytokines, and induce an immune response that causes tumor growth. Therefore, in this study, we investigated the relationship between hematologic parameters and TILs to verify this association. As a result, ALC, AMC, and LMR showed a statistically significant correlation with CD8+ TILs (AMC *r* = 0.22, *p* = 0.010; ALC *r* = − 0.24, *p* = 0.004; LMR *r* = 0.20, *p* = 0.019). However, in a study of esophageal squamous cell carcinoma, CD8+ TILs and AMC showed a negative correlation, and CD8+ TILs and ALC showed a positive correlation with cancer prognosis, which is not consistent with our findings (AMC *r* = − 0.29, *p* <  0.001 ALC *r* = 0.13, *p* = 0.056) [43]. Since there are few studies regarding these associations, more research is needed to make these relationships clear.

Among the hematologic parameters, NLR was the most significant factor in predicting treatment response after NAC, and low NLR showed a tendency toward a better response to NAC (*p* for trend = 0.063). These results may explain the theoretical basis that lymphocytes could lead to the death of cancer cells in response to chemotherapy by presenting tumor-associated antigens to immune cells [44, 45]. Chen et al. and Xu et al. suggested that low NLR could predict a high rate of pCR [46, 47]. However, some studies have shown that NLR was not important as a predictor of pCR after NAC [48, 49].

## Conclusions

We have demonstrated that CD8+ TILs, and hematologic parameters including ALC, AMC, and LMR have the relevance in breast cancer. LMR showed potential as a marker that can strongly predict DFS and OS in breast cancer. However, LMR did not have any value as a prognostic factor in HER^+^ breast cancers. ALC affected LMR in HR^+^ subtype and AMC affected LMR in TN subtype. TILs had different prognostic impacts across breast cancer subtypes, although they were not statistically significant. These results suggest that the influence of the immune system on breast cancer progression may be different depending on the subtype. Therefore, it may be necessary to adopt a different approach depending on the breast cancer subtype in future immune-related studies of this disease.

## Additional files

Additional file 1: **Figure S1.** Kaplan–Meier survival analysis of baseline hematologic parameters (NLR, PLR), TILs CD8+, FOXP3+) in 145 breast cancer patients. (A) DFS curves for NLR (B) PLR (C) CD8+ (D) FOXP3+. NLR, neutrophil-to-lymphocyte ratio; PLR, platelet-to-lymphocyte ratio; FOXP3, forkhead box protein 3. (TIF 1368 kb)

Additional file 2: **Table S1.** Univariate and multivariate Cox regression analyses of TILs, LMR, and clinicopathological characteristics for overall survival in patients with breast cancer. (DOCX 21 kb)

## Abbreviations

ALC: Absolute lymphocyte count
AMC: Absolute monocyte count
ANC: Absolute neutrophil count
DFS: Disease-free survival
EDTA: Ethylenediaminetetraacetic acid
ER: Estrogen receptor
FOXP3: Forkhead box protein 3
HER2: Human epidermal growth factor receptor 2
IHC: Immunohistochemical
LMR: Lymphocyte-to-monocyte ratio
NAC: Neoadjuvant chemotherapy
NLR: Neutrophil-to-lymphocyte ratio
NR: No response
OS: Overall survival
pCR: Pathologic complete response
PD: Progressive disease
PLR: Platelet-to-lymphocyte ratio
PR: Partial response
RECIST: Response Evaluation Criteria in Solid Tumors
SD: Stable disease
TILs: Tumor-infiltrating lymphocytes

## References

[1] Diakos CI, Charles KA, McMillan DC, Clarke SJ (2014). Cancer-related inflammation and treatment effectiveness. Lancet Oncol.

[2] Couzin-Frankel J (2013). Breakthrough of the year 2013. Cancer immunotherapy Science.

[3] Vacchelli E, Aranda F, Eggermont A, Galon J, Sautes-Fridman C, Cremer I (2014). Trial watch: chemotherapy with immunogenic cell death inducers. Oncoimmunology.

[4] Grivennikov SI, Greten FR, Karin M (2010). Immunity, inflammation, and cancer. Cell.

[5] Seretis C, Gourgiotis S, Gemenetzis G, Seretis F, Lagoudianakis E, Dimitrakopoulos G (2013). The significance of neutrophil/lymphocyte ratio as a possible marker of underlying papillary microcarcinomas in thyroidal goiters: a pilot study. Am J Surg.

[6] Walsh SR, Cook EJ, Goulder F, Justin TA, Keeling NJ (2005). Neutrophil-lymphocyte ratio as a prognostic factor in colorectal cancer. J Surg Oncol.

[7] Proctor MJ, McMillan DC, Morrison DS, Fletcher CD, Horgan PG, Clarke SJ (2012). A derived neutrophil to lymphocyte ratio predicts survival in patients with cancer. Br J Cancer.

[8] Lee HE, Chae SW, Lee YJ, Kim MA, Lee HS, Lee BL (2008). Prognostic implications of type and density of tumour-infiltrating lymphocytes in gastric cancer. Br J Cancer.

[9] Nosho K, Baba Y, Tanaka N, Shima K, Hayashi M, Meyerhardt JA (2010). Tumour-infiltrating T-cell subsets, molecular changes in colorectal cancer, and prognosis: cohort study and literature review. J Pathol.

[10] Clemente Claudio G., Mihm Martin C., Bufalino Rosaria, Zurrida Stefano, Collini Paola, Cascinelli Natale (1996). Prognostic value of tumor infiltrating lymphocytes in the vertical growth phase of primary cutaneous melanoma. Cancer.

[11] Dirican A, Ekinci N, Avci A, Akyol M, Alacacioglu A, Kucukzeybek Y (2013). The effects of hematological parameters and tumor-infiltrating lymphocytes on prognosis in patients with gastric cancer. Cancer Biomark.

[12] Mahmoud SM, Paish EC, Powe DG, Macmillan RD, Grainge MJ, Lee AH (2011). Tumor-infiltrating CD8+ lymphocytes predict clinical outcome in breast cancer. J Clin Oncol.

[13] Liu S, Lachapelle J, Leung S, Gao D, Foulkes WD, Nielsen TO (2012). CD8+ lymphocyte infiltration is an independent favorable prognostic indicator in basal-like breast cancer. Breast Cancer Res.

[14] Ladoire S, Mignot G, Dalban C, Chevriaux A, Arnould L, Rebe C (2012). FOXP3 expression in cancer cells and anthracyclines efficacy in patients with primary breast cancer treated with adjuvant chemotherapy in the phase III UNICANCER-PACS 01 trial. Ann Oncol.

[15] Kim MH, Koo JS, Lee S (2013). FOXP3 expression is related to high Ki-67 index and poor prognosis in lymph node-positive breast cancer patients. Oncology.

[16] West NR, Kost SE, Martin SD, Milne K, Deleeuw RJ, Nelson BH (2013). Tumour-infiltrating FOXP3(+) lymphocytes are associated with cytotoxic immune responses and good clinical outcome in oestrogen receptor-negative breast cancer. Br J Cancer.

[17] Iyengar Neil M., Hudis Clifford A., Dannenberg Andrew J. (2013). Obesity and Inflammation: New Insights into Breast Cancer Development and Progression. American Society of Clinical Oncology Educational Book.

[18] Coussens LM, Werb Z (2002). Inflammation and cancer. Nature.

[19] Jiang L, Jiang S, Situ D, Lin Y, Yang H, Li Y (2015). Prognostic value of monocyte and neutrophils to lymphocytes ratio in patients with metastatic soft tissue sarcoma. Oncotarget.

[20] Neofytou K, Smyth EC, Giakoustidis A, Khan AZ, Cunningham D, Mudan S (2014). Elevated platelet to lymphocyte ratio predicts poor prognosis after hepatectomy for liver-only colorectal metastases, and it is superior to neutrophil to lymphocyte ratio as an adverse prognostic factor. Med Oncol.

[21] Jiang L, Zhao Z, Jiang S, Lin Y, Yang H, Xie Z (2015). Immunological markers predict the prognosis of patients with squamous non-small cell lung cancer. Immunol Res.

[22] Porrata LF, Inwards DJ, Ansell SM, Micallef IN, Johnston PB, Hogan WJ (2014). Infused autograft lymphocyte to monocyte ratio and survival in diffuse large B cell lymphoma. Biol Blood Marrow Transplant.

[23] Ni XJ, Zhang XL, Ou-Yang QW, Qian GW, Wang L, Chen S (2014). An elevated peripheral blood lymphocyte-to-monocyte ratio predicts favorable response and prognosis in locally advanced breast cancer following neoadjuvant chemotherapy. PLoS One.

[24] Azab B, Shah N, Radbel J, Tan P, Bhatt V, Vonfrolio S (2013). Pretreatment neutrophil/lymphocyte ratio is superior to platelet/lymphocyte ratio as a predictor of long-term mortality in breast cancer patients. Med Oncol.

[25] Elston CW, Ellis IO (2002). Pathological prognostic factors in breast cancer. I. The value of histological grade in breast cancer: experience from a large study with long-term follow-up. C. W. Elston & I. O. Ellis. Histopathology 1991; 19; 403–410. Histopathology.

[26] Eisenhauer EA, Therasse P, Bogaerts J, Schwartz LH, Sargent D, Ford R (2009). New response evaluation criteria in solid tumours: revised RECIST guideline (version 1.1). Eur J Cancer.

[27] Ghebeh H, Barhoush E, Tulbah A, Elkum N, Al-Tweigeri T, Dermime S (2008). FOXP3+ Tregs and B7-H1+/PD-1+ T lymphocytes co-infiltrate the tumor tissues of high-risk breast cancer patients: implication for immunotherapy. BMC Cancer.

[28] Cimino-Mathews A, Thompson E, Taube JM, Ye X, Lu Y, Meeker A (2016). PD-L1 (B7-H1) expression and the immune tumor microenvironment in primary and metastatic breast carcinomas. Hum Pathol.

[29] Seo AN, Lee HJ, Kim EJ, Kim HJ, Jang MH, Lee HE (2013). Tumour-infiltrating CD8+ lymphocytes as an independent predictive factor for pathological complete response to primary systemic therapy in breast cancer. Br J Cancer.

[30] Hornychova H, Melichar B, Tomsova M, Mergancova J, Urminska H, Ryska A (2008). Tumor-infiltrating lymphocytes predict response to neoadjuvant chemotherapy in patients with breast carcinoma. Cancer Investig.

[31] Liu F, Lang R, Zhao J, Zhang X, Pringle GA, Fan Y (2011). CD8(+) cytotoxic T cell and FOXP3(+) regulatory T cell infiltration in relation to breast cancer survival and molecular subtypes. Breast Cancer Res Treat.

[32] Asano Y, Kashiwagi S, Goto W, Kurata K, Noda S, Takashima T (2016). Tumour-infiltrating CD8 to FOXP3 lymphocyte ratio in predicting treatment responses to neoadjuvant chemotherapy of aggressive breast cancer. Br J Surg.

[33] Whiteside TL (2010). Immune responses to malignancies. J Allergy Clin Immunol.

[34] Ownby HE, Roi LD, Isenberg RR, Brennan MJ (1983). Peripheral lymphocyte and eosinophil counts as indicators of prognosis in primary breast cancer. Cancer.

[35] Ray-Coquard I, Cropet C, Van Glabbeke M, Sebban C, Le Cesne A, Judson I (2009). Lymphopenia as a prognostic factor for overall survival in advanced carcinomas, sarcomas, and lymphomas. Cancer Res.

[36] Ceze N, Thibault G, Goujon G, Viguier J, Watier H, Dorval E (2011). Pre-treatment lymphopenia as a prognostic biomarker in colorectal cancer patients receiving chemotherapy. Cancer Chemother Pharmacol.

[37] Rashid F, Waraich N, Bhatti I, Saha S, Khan RN, Ahmed J (2010). A pre-operative elevated neutrophil: lymphocyte ratio does not predict survival from oesophageal cancer resection. World J Surg Oncol.

[38] Lord BI, Bronchud MH, Owens S, Chang J, Howell A, Souza L (1989). The kinetics of human granulopoiesis following treatment with granulocyte colony-stimulating factor in vivo. Proc Natl Acad Sci U S A.

[39] Steidl C, Lee T, Shah SP, Farinha P, Han G, Nayar T (2010). Tumor-associated macrophages and survival in classic Hodgkin's lymphoma. N Engl J Med.

[40] Peterson JE, Zurakowski D, Italiano JE, Michel LV, Connors S, Oenick M (2012). VEGF, PF4 and PDGF are elevated in platelets of colorectal cancer patients. Angiogenesis.

[41] Takeuchi H, Kawanaka H, Fukuyama S, Kubo N, Hiroshige S, Yano T (2017). Comparison of the prognostic values of preoperative inflammation-based parameters in patients with breast cancer. PLoS One.

[42] Jia W, Wu J, Jia H, Yang Y, Zhang X, Chen K (2015). The peripheral blood neutrophil-to-lymphocyte ratio is superior to the lymphocyte-to-monocyte ratio for predicting the long-term survival of triple-negative breast Cancer patients. PLoS One.

[43] Zhu Y, Li M, Bo C, Liu X, Zhang J, Li Z (2017). Prognostic significance of the lymphocyte-to-monocyte ratio and the tumor-infiltrating lymphocyte to tumor-associated macrophage ratio in patients with stage T3N0M0 esophageal squamous cell carcinoma. Cancer Immunol Immunother.

[44] Youn JI, Collazo M, Shalova IN, Biswas SK, Gabrilovich DI (2012). Characterization of the nature of granulocytic myeloid-derived suppressor cells in tumor-bearing mice. J Leukoc Biol.

[45] Apetoh L, Ghiringhelli F, Tesniere A, Obeid M, Ortiz C, Criollo A (2007). Toll-like receptor 4-dependent contribution of the immune system to anticancer chemotherapy and radiotherapy. Nat Med.

[46] Chen Y, Chen K, Xiao X, Nie Y, Qu S, Gong C (2016). Pretreatment neutrophil-to-lymphocyte ratio is correlated with response to neoadjuvant chemotherapy as an independent prognostic indicator in breast cancer patients: a retrospective study. BMC Cancer.

[47] Xu J, Ni C, Ma C, Zhang L, Jing X, Li C (2017). Association of neutrophil/lymphocyte ratio and platelet/lymphocyte ratio with ER and PR in breast cancer patients and their changes after neoadjuvant chemotherapy. Clin Transl Oncol.

[48] Eryilmaz MK, Mutlu H, Salim DK, Musri FY, Tural D, Coskun HS (2014). The neutrophil to lymphocyte ratio has a high negative predictive value for pathologic complete response in locally advanced breast cancer patients receiving neoadjuvant chemotherapy. Asian Pac J Cancer Prev.

[49] Adachi K, Sakurai K, Suzuki S, Hara Y, Nagashima S, Hirano T (2015). Study of the response rate and neutrophil lymphocyte ratio in breast Cancer patients undergoing neoadjuvant chemotherapy. Gan To Kagaku Ryoho.

