# Supplementary figures and images for: The prognostic and predictive value of tumor-infiltrating lymphocytes and hematologic parameters in patients with breast cancer
Source: BMC Cancer. 2018 Oct 1;18:938. doi: 10.1186/s12885-018-4832-5 (PMC6167816; doi:10.1186/s12885-018-4832-5)

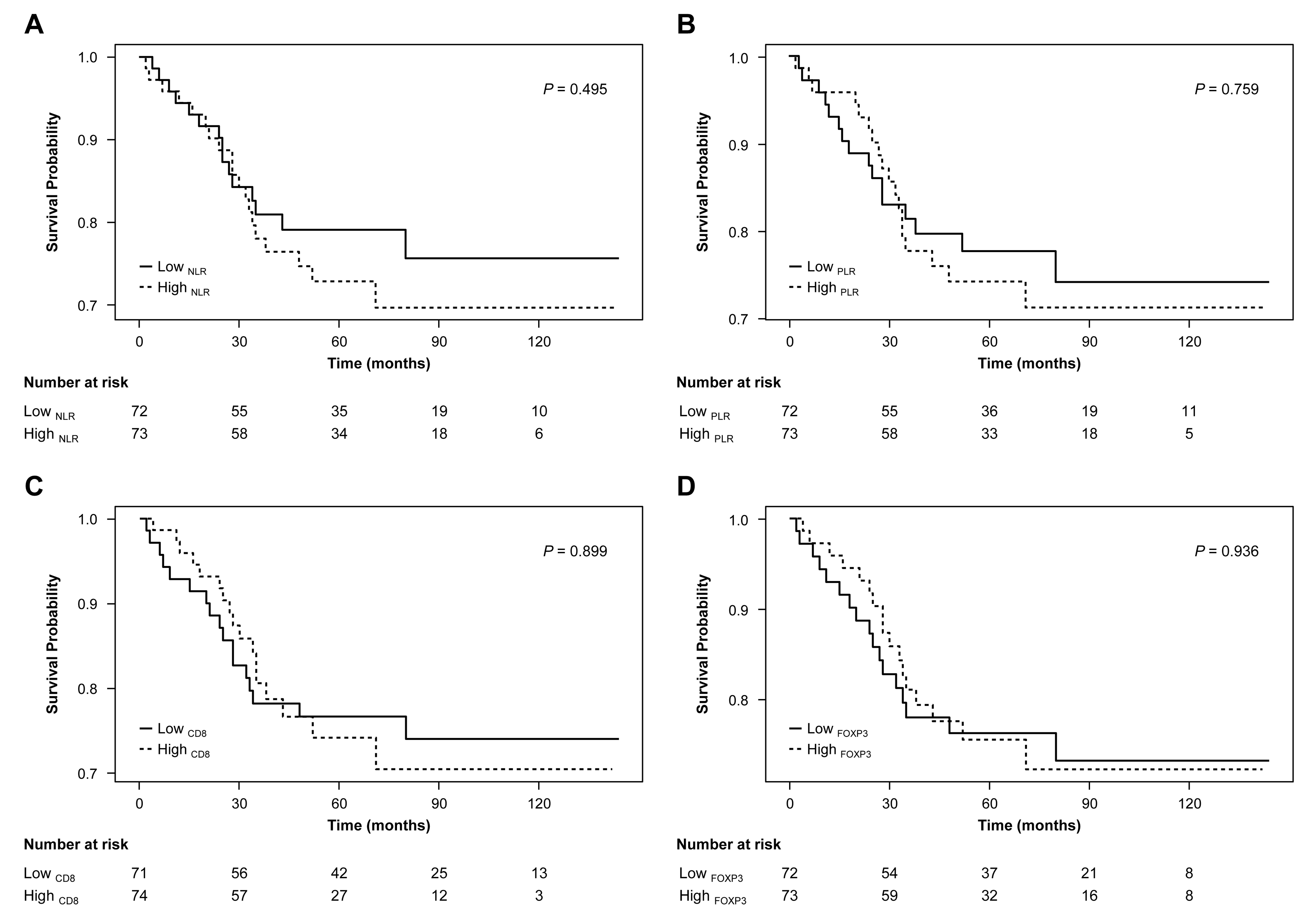

Supplement: Supplementary file 1 — Figure S1. Kaplan–Meier survival analysis of baseline hematologic parameters (NLR, PLR), TILs CD8+, FOXP3+) in 145 breast cancer patients. (A) DFS curves for NLR (B) PLR (C) CD8+ (D) FOXP3+. NLR, neutrophil-to-lymphocyte ratio; PLR, platelet-to-lymphocyte ratio; FOXP3, forkhead box protein 3. (TIF 1368 kb) [file 12885_2018_4832_MOESM1_ESM.tif]
