# Supplementary material for: The prognostic and predictive value of tumor-infiltrating lymphocytes and hematologic parameters in patients with breast cancer
Source: BMC Cancer. 2018 Oct 1;18:938. doi: 10.1186/s12885-018-4832-5 (PMC6167816; doi:10.1186/s12885-018-4832-5)
Supplement: Supplementary file 2 — Table S1. Univariate and multivariate Cox regression analyses of TILs, LMR, and clinicopathological characteristics for overall survival in patients with breast cancer. (DOCX 21 kb) [file 12885_2018_4832_MOESM2_ESM.docx]

**Table S1. Univariate and multivariate Cox regression analyses of TILs, LMR, and clinicopathological characteristics for overall survival in patients with breast cancer**

| **Variable** | **Disease-free survival** | | | | |
| --- | --- | --- | --- | --- | --- |
|  | **Univariate analysis** | |  | **Multivariate analysis** | |
|  | **HR (95% CI)** | ***P*** |  | **HR (95% CI)** | ***P*** |
| Age (>50 *vs.* ≤50) | 0.77 (0.24-2.43) | 0.658 |  |  |  |
| T stage (>T1 *vs.* T1) |  |  |  |  |  |
| N stage (>N0 *vs.* N0) | 7.89 (1.02-61.29) | 0.048 |  | 7.12(0.88-57.41) | 0.065 |
| Histologic grade (G3 *vs.* <G3) | 1.29 (0.41-4.06) | 0.667 |  |  |  |
| Lymphovascular invasion (yes *vs.* no) | 4.60 (1.24-16.99) | 0.022 |  | 3.65(0.96-13.84) | 0.057 |
| Estrogen receptor (positive *vs.* negative) | 0.36 (0.11-1.21) | 0.100 |  | 0.39 (0.12-1.35) | 0.138 |
| Progesterone receptor(positive *vs.* negative) | 0.46 (0.12-1.70) | 0.242 |  |  |  |
| HER2 (positive *vs.* negative) | 0.62 (0.14-2.85) | 0.544 |  |  |  |
| Type of operation (Mastectomy *vs.* BCS) |  |  |  |  |  |
| CD8 (high *vs.* low) | 0.35 (0.09-1.29) | 0.115 |  | 0.36 (0.09-1.43) | 0.147 |
| FOXP3 (high *vs.* low) | 0.32 (0.09-1.19) | 0.089 |  | 0.38 (0.09-1.52) | 0.170 |
| LMR (high *vs.* low) | 0.17 (0.04-0.78) | 0.022 |  | 0.17(0.04-0.80) | 0.025 |
| Patients with T1 tumor or BCS did not die. *CI* confidence interval, *HER2* human epidermal growth factor receptor 2, *FOXP3* forkhead box protein 3, *BCS* breast conserving surgery, *LMR*, lymphocyte/monocyte ratio. | | | | | |
